# Separate multisensory integration processes for ownership and localization of body parts

**DOI:** 10.1038/s41598-018-37375-z

**Published:** 2019-01-24

**Authors:** Kazumichi Matsumiya

**Affiliations:** 1 Graduate School of Information Sciences, Tohoku University, 6-3-09 Aoba, Aramaki-aza, Aoba-ku, Sendai 980-8579 Japan; 2 Japan Science and Technology Agency, Precursory Research for Embryonic Science and Technology (PRESTO), 6-3-09 Aoba, Aramaki-aza, Aoba-ku, Sendai 980-8579 Japan

## Abstract

The experiences that body parts are owned and localized in space are two key aspects of body awareness. Although initial work assumed that the perceived location of one’s body part can be used as a behavioral measure to assess the feeling of owning a body part, recent studies call into question the relationship between localization and ownership of body parts. Yet, little is known about the processes underlying these two aspects of body-part awareness. Here, I applied a statistically optimal cue combination paradigm to a perceptual illusion in which ownership over an artificial hand is experienced, and found that variances predicted by a model of optimal cue combination are similar to those observed in localization of the participant’s hand, but systematically diverge from those observed in ownership of the artificial hand. These findings provide strong evidence for separate processes between ownership and localization of body parts, and indicate a need to revise current models of body part ownership. Results from this study suggest that the neural substrates for perceptual identification of one’s body parts—such as body ownership—are distinct from those underlying spatial localization of the body parts, thus implying a functional distinction between “who” and “where” in the processing of body part information.

## Introduction

When we see our body parts, such as our hands, we easily feel that they are part of our own body. This feeling is referred to as body ownership^1–4^. It has been suggested that body ownership can be seen as the multisensory perception of one’s own body^1,2,5^. The spatial and temporal congruence of visual, tactile, and proprioceptive signals from one’s body part generates a feeling of ownership for the body part. Though the feeling of body ownership is one of the most important features of self-consciousness^6,7^, the issue of precisely how we experience body parts to be part of the self is a fundamental question in cognitive science and neuroscience. This problem is biologically relevant, as the conscious experience of our own body parts is essential for our interactions with the outside world. A seminal study in this field described a simple procedure to induce the feeling of ownership of a rubber hand, known as the rubber hand illusion (RHI)^8^, which has been used as a model system for investigating the feeling of body ownership. In this illusion, the observer’s real hand is hidden from view, while a realistic life-sized rubber hand is presented in front of the observer and is placed several centimeters away from the observer’s hidden hand. The experimenter uses two paintbrushes, with one stroking the rubber hand and the other stroking the observer’s hidden hand, with the timing of the brushing synchronized. After a time, the rubber hand is felt to be one’s own, and the perceived location of the real hand is displaced toward the rubber hand—a phenomenon known as proprioceptive drift. The original study evaluated the RHI using two measures^8^: a subjective measure and an objective one. The subjective measure comprised a questionnaire with visual analogue scales, such as “I felt as if the rubber hand was my own hand”. The objective measure was constructed by perceived location of the real hand with the observer’s eyes closed. The study found that substantial displacement of the perceived location of the real hand toward the rubber hand (i.e., large proprioceptive drift) occurred during the synchronous stroking of the real and rubber hands, compared with when stroking was asynchronous. Furthermore, it indicated that the magnitude of the proprioceptive drift correlated with the strength of the feeling of ownership reported in the questionnaire.

Initial work assumed that proprioceptive drift can be used as a behavioral measure to assess the subjective feeling of body ownership^4,8^, but recent studies call into question the relationship between the two^9–11^. In fact, Rohde *et al*.^11^ reported that proprioceptive drift can occur without a subjective feeling of hand ownership^11^. Proprioceptive drift reflects the experience of where one’s body parts are perceived to be located in space, while the subjective feeling of ownership of body parts reflects the experience of identifying with the body parts. These aspects may depend on distinct neural processes^12^. Yet, very little is known about the processes underlying two of these aspects of one’s own body parts.

Characteristics of the constraints of the RHI provide important information about the factors relevant for body ownership. A temporal mismatch of 500 ms between vision and touch, such as in the asynchronous stroking of the real and rubber hands, reduces the strength of the RHI^13^. The RHI is also limited by the spatial distance between the real and rubber hands^14^. A significant decrease in the strength of the RHI has been found for distances greater than 30 cm. These temporal and spatial constraints fit well with the principles of the integration of multiple sources of sensory information (multisensory integration)^1^. Thus, the illusion is generally explained in terms of the integration of visual, tactile, and proprioceptive information, although a recent study has suggested that the illusion can occur based on visual-proprioceptive integration even without tactile stimulation^5^.

In the present study, I used a RHI in which ownership over a computer graphics (CG) hand is experienced^15,16^, and applied a statistically optimal cue combination paradigm^17,18^ to the illusion. The optimal cue combination paradigm is used to investigate the processes underlying multisensory integration. This paradigm provides a way to determine the degree to which a given sensory modality contributes to the final perception relative to a different sensory modality. This is realized by using maximum likelihood estimation (MLE) to combine multiple sensory inputs^19^. To construct a maximum-likelihood integrator, variances associated with sensory estimation are used. In the present study, I measured variances associated with visual and proprioceptive estimation of hand position. These measurements were used to construct a model of optimal integration of visual and proprioceptive information. Then, I measured the perceived location of the participant’s hand and the sense of ownership over the CG hand in the RHI tasks. These measurements were compared to the behavior of the model of optimal integration of visual and proprioceptive information. I found that perceived location of the participant’s hand in the RHI is well explained by the model of optimal integration of visual and proprioceptive information, but that sense of ownership is not. These findings reveal different visual-proprioceptive integration processes for localization of body parts and ownership of body parts.

## Results

### Experiment 1. Visual-alone condition and proprioceptive-alone condition

#### Procedure

**Figure 1:**
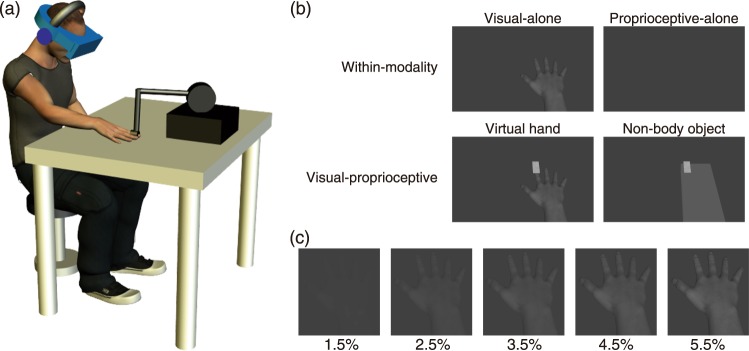
Apparatus and stimuli. (**a**) Participants binocularly viewed the visual stimulus presented on a head-mounted display, and put their right hand on a table. Their right index finger was attached to the arm of a force-feedback device. (**b**) Top row, the left and right panels represent visual stimuli for the visual-alone and proprioceptive-alone conditions, respectively. Bottom row, the left and right panels show a computer graphics (CG) hand and a CG non-hand object used in the visual-proprioceptive condition, respectively. (**c**) The contrast of the CG hand was selected randomly between 1.5%, 2.5%, 3.5%, 4.5%, and 5.5% from trial to trial.

I examined the variances of visual and proprioceptive hand-position estimates (within-modality hand localization). Participants were asked to localize either a realistic life-sized CG hand or their own hidden hand, presented unimodally. In the visual-alone condition, the moving CG hand, which was a grayscale three-dimensional visual image of various luminance contrasts, was presented through a head-mounted display (HMD; Fig. 1). In the proprioceptive-alone condition, the participant’s entire right arm, which was hidden, was moved by the arm of a force-feedback device attached to the participant’s right index finger. I obtained the proportion of trials in which the location where the hand turned back in the first hand movement was perceived to be to the right of the location where the hand turned back in the second hand movement, as a function of the actual physical location of the turn (2.5–7.5 cm). By using Probit analysis^20^, the data were fitted with Gaussian cumulative distribution functions, which provided the standard deviation of the fitted Gaussian cumulative distribution function. The mean of the fitted function represents the point of subjective equality (the 50% criterion value). The standard deviation of the fitted function represents the estimate of localization accuracy, which is presumed to depend on internal noise.

#### Results

**Figure 2:**
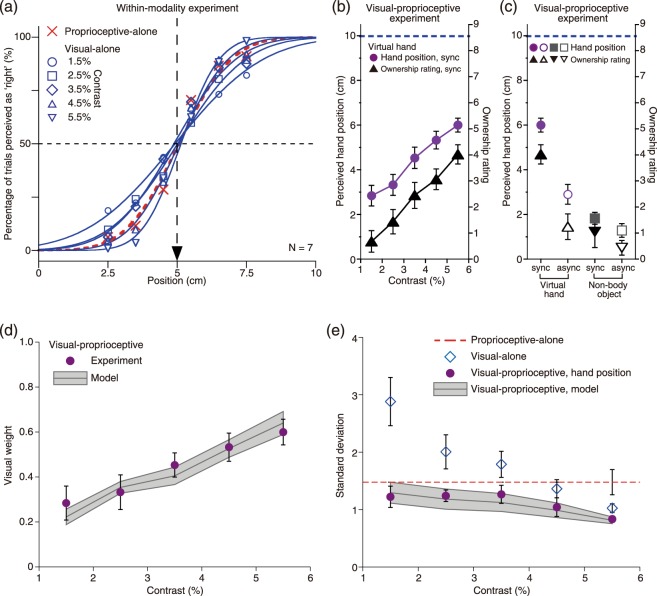
Results. (**a**) Results for within-modality experiments. Psychometric functions for localizing either hand proprioception or CG hands of various contrasts. Data were averaged for participants. Turned position of the standard stimulus was 5 cm to the right of the median plane of the participant’s body. Proprioceptive discrimination data are represented by the red curve. Visual discrimination data are represented by blue curves, which correspond to five contrasts of the CG hand. (**b**) Results for the visual-proprioceptive condition when the CG hand was presented in the synchronous stroking between vision and touch. Abscissa represents the contrast of the CG hand, left ordinate represents perceived position of the participant’s hand relative to their unseen hand (purple circles), and right ordinate represents subjective rating of ownership of the CG hand (black triangles). The dashed line represents the location of the CG hand relative to the participant’s unseen hand. (**c**) Results for the CG hand and the non-body object for synchronous and asynchronous stroking between vision and touch. Solid and open symbols represent synchrony and asynchrony, respectively. The contrasts of the CG hand and the non-body object were 5.5% for both the synchrony and asynchrony conditions. (**d**) Visual weights as a function of contrast of the CG hand. The shaded area represents predicted visual weights expected from within-modality discrimination, and its height represents predicted errors given the standard errors of the within-modality discrimination. Purple symbols represent observed visual weights obtained from Eq. 3 using the values of perceived hand positions. Visual weights for the CG hand with asynchronous stroking between vision and touch and visual weights for the non-body object are shown in Fig. S2. (**e**) Standard deviations as a function of contrast of the CG hand. The shaded area represents predicted standard deviations (Eq. 4). Purple symbols represent obtained standard deviations of perceived hand position in the rubber hand illusion tasks. Blue open square symbols represent the visual-alone standard deviations, and the dashed red line represents the proprioceptive-alone standard deviation. *n* = 7. Results are means ± standard error of the mean.

Discrimination results for visual-alone and proprioceptive-alone conditions are shown in Fig. 2a. The point of subjective equality was near 5 cm in both of the conditions, but the standard deviation varied considerably. The standard deviation for the visual-alone condition decreased from 2.88 to 1.03, as the contrast of the CG hand was increased from 1.5% to 5.5%. For the proprioceptive-alone condition, the standard deviation was 1.48: falling midway between the various standard deviations for the visual-alone condition.

### Experiment 2. Visual-proprioceptive condition: RHI

#### Procedure

To examine the strength of the RHI using a CG hand, I measured the perceived position of the participant’s own unseen hand while looking at a CG hand with stroking of both the CG hand and the participant’s unseen hand. In the visual-proprioceptive condition, the contrast of the CG hand was varied randomly from trial to trial (0.5–5.5%). The CG hand was positioned 10 cm to the left of the participant’s unseen right hand (Δ = S_P_ − S_V_ = 10 cm, where S_V_ and S_P_ are the hand positions from the visual and proprioceptive cues). The average of S_V_ and S_P_ was 5 cm. The participant’s own unseen hand was stroked for 60 s, with synchronous stroking of the CG hand (or with asynchronous stroking in the control condition; see below). Next, participants were asked to point at their own unseen right hand by moving a visual pointer with their left hand. After the hand localization task, participants were asked to subjectively rate their ownership of the CG hand using a nine-point scale (hand ownership task; see methods for details).

#### Results

Figure 2b shows the strength of the RHI as a function of the contrast of the CG hand. The mean perceived position of the real hand with synchronous stroking of the CG and real hands depended significantly on CG hand contrast (F_4,24_ = 19.71, *p* < 0.0001). For the low-contrast CG hands, the perceived position of the real hand was near the hand location provided by the proprioceptive information, not by the visual information, suggesting that proprioception contributes to the perceived position of the real hand to a greater degree than does vision. However, for the high-contrast CG hands, the opposite pattern was observed with the perceived position of the real hand near the hand location provided by the visual information, suggesting that vision contributes to perceived position more than proprioception does. For the mid-contrast CG hand, the perceived position was the average position of the two modalities. The subjective ratings of ownership of the CG hand also had the same tendency to the perceived position of the real hand (Fig. 2b; F_4,24_ = 23.24, *p* < 0.0001). It has been previously shown that the magnitude of proprioceptive drift correlates with the strength of the feeling of ownership reported in a questionnaire, except for when special condition to induce RHI are used, such as in Rohde *et al*.’s study^11^. Thus, the finding that the perceived position and the ownership rating are similarly affected by the contrast of the CG hand is consistent with the results of previous research^8^.

### Experiment 3. Control conditions for the RHI

#### Procedure

I also conducted control experiments to confirm the occurrence of RHI in the present study. RHI has been reported to be eliminated in the following conditions: (i) asynchronous stroking of the CG hand and the real hand and (ii) presentation of a non-body object (Fig. 1b) instead of the CG hand for both synchronous and asynchronous stroking between vision and touch. A fixed 5.5% contrast was chosen for the CG hand or the non-body object in these control conditions. In Experiment 2, the highest contrast of the CG hand was 5.5% (original condition). If the RHI occurred in the present study, the illusion should be eliminated even with the highest contrast of 5.5%. Therefore, using this fixed 5.5% contrast, I compared the strength of the RHI between the original and control conditions.

#### Results

Figure 2c shows the strength of the RHI in the original and control conditions. The perceived position of the real hand and the subjective ratings of ownership depended significantly on condition (F_3,24_ = 21.53, *p* < 0.0001 for perceived position; F_3,24_ = 12.12, *p* < 0.0005 for ownership). When the stroking of the CG hand and the real hand was asynchronous, the perceived position of the real hand was not greatly displaced toward the CG hand’s position, even for the high contrast. Similarly, when the non-body object was presented with synchronous stroking of the object and the real hand, the perceived position of the real hand was not greatly displaced toward the object’s position. Compared with synchronous stroking, ownership rating decreased when the stroking of the CG hand and the real hand was asynchronous or when the non-body object was presented (irrespective of synchronous or asynchronous stroking of the object and the real hand). These results indicate that synchronized visual and tactile stimulation with the CG hand causes the RHI, which is consistent with the results of previous research^8,21–23^.

### Maximum-likelihood estimation

#### Method

Several studies have suggested that multimodal information may be combined in a fashion similar to a MLE integration process, with a final estimate being obtained by summing the independent estimates from each modality according to an appropriate weighting scheme^17–19^. Assuming that the estimate from each modality is corrupted by independent Gaussian noise, the weights are proportional to the inverse of the variance of the noise distribution. This model allows the optimal combinations of the estimates from different modalities because the final estimate from combining them has lower variance than the estimates from each modality. If the model is used to combine visual and proprioceptive estimates ${\hat{S}}_{V}$ and ${\hat{S}}_{P}$, the final estimate ${\hat{S}}_{VP}$ is given by

1 $${\hat{S}}_{VP}={w}_{V}{\hat{S}}_{V}+{w}_{P}{\hat{S}}_{P}$$

where ${w}_{V}$ and ${w}_{P}$ are the relative weights for each modality. The weights ${w}_{V}$ and ${w}_{P}$ are given by

2 $${w}_{V}=\frac{1/{\sigma }_{V}^{2}}{1/{\sigma }_{V}^{2}+1/{\sigma }_{P}^{2}}=\frac{{\sigma }_{P}^{2}}{{\sigma }_{V}^{2}+{\sigma }_{P}^{2}}\,{\rm{and}}\,{w}_{P}=\frac{1/{\sigma }_{P}^{2}}{1/{\sigma }_{V}^{2}+1/{\sigma }_{P}^{2}}=\frac{{\sigma }_{V}^{2}}{{\sigma }_{V}^{2}+{\sigma }_{P}^{2}}$$

where ${\sigma }_{V}$ and ${\sigma }_{P}$ are the visual and proprioceptive standard deviations.

Estimates of the visual and proprioceptive standard deviations can be obtained by using the standard deviation of the cumulative Gaussian function fitted to the within-modality data (Fig. 2a). From Eq. 2 and these estimates, visual and proprioceptive weights can be predicted for the various contrasts of the CG hand. Now, I assume that the visual and proprioceptive estimates (${\hat{S}}_{V}$ and ${\hat{S}}_{P}$) are given by the actual position of the visual and proprioceptive sources (${S}_{V}$ and ${S}_{P}$), and that the sum of the visual and proprioceptive weights is 1. Following these assumptions, the visual weights can be experimentally derived from the visual-proprioceptive data (hand position in Fig. 2b) using Eq. 1:

3 $$\begin{array}{c}{w}_{V}=({\rm{P}}{\rm{P}}-{S}_{P})/({S}_{V}-{S}_{P})\\ (\,\because {\hat{S}}_{VP}={\rm{P}}{\rm{P}})\end{array}$$

where PP is the perceived position of the real hand when looking at the CG hand with stroking of both the real and CG hands.

Furthermore, the MLE integration model predicts that the standard deviation of the combined estimate is lower than that of either the visual or proprioceptive estimate alone. According to the model, the standard deviation ${\sigma }_{VP}$ of the combined estimate is calculated using the formula:

4 $${\sigma }_{VP}=\frac{{\sigma }_{V}{\sigma }_{P}}{\sqrt{{\sigma }_{V}^{2}+{\sigma }_{P}^{2}}}\le \,{\rm{\min }}({\sigma }_{V},{\sigma }_{P})$$

where ${\sigma }_{V}$ and ${\sigma }_{P}$ are the visual and proprioceptive standard deviations.

The present study derived estimates of ${\sigma }_{V}$ and ${\sigma }_{P}$ from the visual-alone and proprioceptive-alone localization tasks, respectively. From these, I calculated the combined estimate of ${\sigma }_{VP}$; thus, the prediction was made for hand localization only. The data from the hand localization task in Experiment 2 were used to derive visual weights experimentally. Moreover, the data from the hand localization task and the hand ownership task in Experiment 2 were used to experimentally derive standard deviations for hand localization and hand ownership, respectively. I then compared the predicted noise distribution for hand localization to noise distributions for hand localization and hand ownership in the RHI tasks. If the processing pathway is shared between hand localization and hand ownership in the RHI, these processes should be affected by the same source of noise. If this is true, the predicted noise distribution should agree with the observed noise distributions of both hand localization and hand ownership in the RHI tasks. Because the unit of the standard deviation of hand localization is different from that of hand ownership, I used the coefficient of variation (*CV*) for the comparison between the noise distributions of hand localization and ownership in the RHI tasks. This index is given by

5 $$CV=\frac{\sigma }{\bar{x}}$$

where $\sigma$ is the standard deviation, and $\bar{x}$ is the mean. The *CV* is a dimensionless quantity, which allows a direct comparison between hand localization and hand ownership. The mean and standard deviation of observed perceived positions were used to calculate the *CV* for experimental hand localization for each contrast of the CG hand. In the same way, the mean and standard deviation of observed ownership ratings were used to calculate the *CV* for experimental hand ownership for each contrast of the CG hand. In contrast, to calculate the *CV* for the prediction of hand localization from the model, the mean position of the combined visual-proprioceptive estimate for each contrast was computed using Eq. 1 and the weights derived from the visual-alone and proprioceptive-alone conditions. For each contrast, the predicted standard deviation divided by the mean position gave the *CV* for the predicted hand localization. Note that since the *CV* informs relative comparison, the comparison by *CV* reflects qualitative differences.

#### Results

In Fig. 2d, the predicted visual weights are represented by the curve and the shaded area, and the experimental visual weights are represented by the purple symbols. The predicted visual weights varied with the contrast of the CG hand: visual weights were higher when contrast was high, and lower when contrast was low. The experimental visual weight increased with the contrast of the CG hand, and clearly fell very close to the predicted visual weight. The predicted and experimental standard deviations for the unimodal and multimodal localization tasks are shown in Fig. 2e. Figure 2e shows that the experimentally obtained standard deviations of the perceived location of the participant’s hand in the RHI task followed the predictions of the model. Furthermore, as shown in Fig. 2e, the experimental standard deviations were always lower than the visual-alone and proprioceptive-alone standard deviations across all contrasts of the CG hand, which is consistent with the MLE prediction given by Eq. 4.

**Figure 3:**
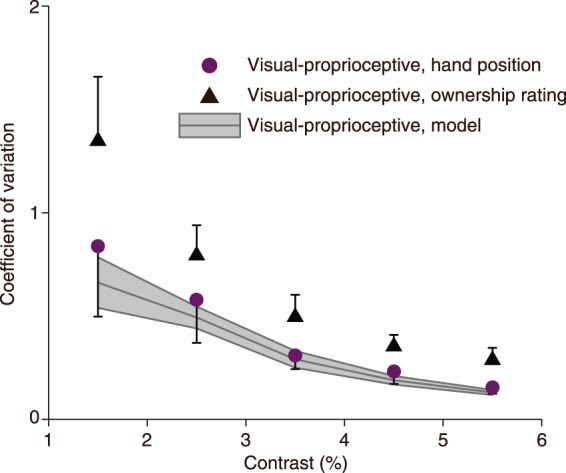
Coefficient of variation (CV) as a function of contrast of the CG hand. The shaded area represents CVs predicted by the maximum likelihood estimation model. Purple and black symbols represent obtained CVs of perceived hand position and hand ownership in the rubber hand illusion tasks, respectively.

Figure 3 shows *CV*s for localization, ownership, and the MLE prediction. In this figure, the experimentally obtained *CV* for localization in the RHI followed the prediction of the MLE prediction. However, the *CV* for ownership in the RHI did not follow the prediction. Statistical analysis revealed that the predicted *CV* was significantly different from the experimental *CV* for ownership (*F*_4,48_ = 6.13, *p* < 0.05), but not from the experimental *CV* for localization (*F*_4,48_ = 0.22, *p* = 0.65 n.s.).

## Discussion

The present results show that the perceived location of the participant’s hand in the RHI is determined by combining visual and proprioceptive information in a manner consistent with the MLE integration process. Yet, the present results indicate that the sense of hand ownership systematically diverges from the predictions of the MLE integration process. Although recent studies have cast doubt on the presumed link between the feeling of ownership over a body part and the perceived location of a body part^9–11^, these studies did not show that the processes underlying these two aspects are dissociated even when strong effects of the RHI occurs. However, an early neuroimaging study has suggested that body ownership is associated with activity in the premotor cortex and that body localization is associated with activity in the posterior parietal cortex^24^. The present results provide strong behavioral evidence that separate mechanisms of multisensory integration underlie ownership and localization of body parts.

MLE and Bayesian causal inference (BCI) models have been shown to account for multisensory perception of the environment^25^. In the MLE model, sensory stimuli are integrated from unisensory stimuli into a single multisensory percept. This model has been demonstrated to apply across audio-visual, visual-haptic, visual-proprioceptive, and visual-vestibular integration. In the BCI model, however, sensory likelihoods are combined with a prior, which makes an inference about the causal structure of events in the environment. The BCI model can determine whether sensory signals are caused by the same source or by different sources. This model has also been demonstrated to account for certain well-known multisensory illusions, such as the ventriloquist illusion and the sound-induced flash illusion^26,27^.

These models have made much progress in our understanding of computational rules of multisensory perception of the environment^25^. However, very little work has been done on the use of computational models of body ownership. Samad *et al*.^5^ provided the first computational account of the RHI by a BCI model^5^. The BCI model explained that synchronous stroking of a dummy hand and a real hand produces the perception of a common cause for visual and tactile stimuli, inducing the RHI. Interestingly, this model predicted that the RHI can occur based on visual-proprioceptive integration absent tactile stimulation, which Samad *et al*. confirmed experimentally. The present study as well indicated that integration of visual and proprioceptive information is used for the RHI, which is consistent with Samad *et al*.’s finding that visual-proprioceptive integration is critical for the RHI.

Although Samad *et al*. showed that the process of the RHI can be modeled as a BCI^5^, they did not examine whether this process can also be modeled by using the MLE rule. The present study indicated that the MLE model can account for hand localization in the RHI. The MLE rule states that visual and proprioceptive information is combined in an optimal way by summing the independent stimulus estimates from each modality, weighted by the inverse of the noise associated with each estimate as given by the variance of the underlying noise distribution. According to this weighting scheme, the stimulus estimate with lower variance is more reliable. The present study manipulated the contrast of the CG hand to adjust the amount of noise in the visual stimulus, and found that reducing the contrast of the CG hand resulted in less reliable visually specified hand location. To elucidate the predictions of the MLE rule, the present study determined the standard deviations of the visual and proprioceptive estimates by conducting unisensory (visual-alone and proprioceptive-alone) location discrimination experiments and derived the predicted standard deviations of the MLE model. Then, these predicted standard deviations were compared to the observed standard deviations of hand localization in the multisensory (visual-proprioceptive) tasks. The results indicate that hand localization in the RHI is well explained by the MLE model of optimal integration of visual and proprioceptive information.

Initial work has assumed a shared process between hand localization and hand ownership in the RHI^4,8^. However, recent studies have cast doubt on the presumed link between them^9–11^. An early neuroimaging study suggested that hand ownership and hand localization were represented via distinct brain areas^24^. Nevertheless, Samad *et al*.’s^5^ previous modeling study did not consider whether a visual-proprioceptive process is shared between hand localization and hand ownership in the RHI^5^. To address this issue, the present study used the MLE model. If the processing pathway is shared between hand localization and hand ownership, these processes should be affected by the same source of noise. To this end, the present study compared the observed standard deviations of hand localization and hand ownership in the multisensory tasks (i.e. the RHI tasks) to the predicted standard deviations of the MLE model from the unisensory localization tasks by using the *CV* as an index. Note that the unisensory ownership experiments were not conducted in the present study. The present results indicate that the predicted *CV*s agree with the observed *CV*s in only the multisensory localization task, but not those in the multisensory ownership task. This finding suggests that the MLE model can account for hand localization in the RHI, but not for hand ownership. In contrast, Samad *et al*.^5^ showed that the BCI model can account for the subjective feeling of hand ownership in the RHI^5^. Taken together, these results suggest a computational account of separate processes between hand localization and hand ownership in the RHI.

What are the underlying neural mechanisms for separate processes between hand localization and hand ownership? Recent neuroimaging work has shown that multisensory integration is governed by distinct computational principles across the cortical hierarchy^28,29^ (for reviews, see Noel *et al*.^25^). At the bottom of the cortical hierarchy, sensory signals are processed in each unisensory area. At the next stage, sensory signals are combined as predicted by the MLE model in the posterior intraparietal sulcus. At the top of the hierarchy, sensory signals are combined as predicted by the BCI model in the anterior intraparietal sulcus. These findings suggest that the unisensory estimates, MLE, and BCI may be represented with distinct brain areas. Although these neuroimaging studies examined multisensory perception of the outside world but not that of one’s own body, I speculate that MLE and BCI may be represented with distinct brain areas for multisensory integration of bodily signals as well as sensory signals from the outside world. Thus, these findings suggest that the neural substrates for perceptual identification of one’s body parts (body ownership) may be distinct from those underlying spatial localization of the body parts.

The present study revealed a qualitative difference between hand localization and hand ownership using the MLE model. However, a quantitative examination would be needed in order to develop a neurobiological model of body ownership. The present study did not apply the MLE model to hand ownership, due to the seeming impossibility of estimating hand ownership in unisensory conditions by using stimuli to induce the RHI. For example, in a visual-alone condition, the CG hand would never be felt to be one’s own hand, regardless of the contrast. In a proprioceptive-alone condition, participants would constantly feel their own hand when it is hidden from view. Future research would need to implement a new method in order to acquire estimates of hand ownership in unisensory conditions.

How do the present findings apply to whole-body ownership? A neuroimaging study has suggested that whole-body ownership is produced by neuronal populations that integrate multisensory information across body parts^30^. Recent behavioral studies have also suggested that most body parts are anatomically connected to the trunk^31,32^. Furthermore, a recent neuroimaging study has shown that self-location is associated with parieto-cingulate-hippocampal activity, whereas whole-body ownership is associated with premotor-intraparietal activity^33^. This indicates that whole-body ownership and self-location are represented with distinct neural populations at the early stages of whole-body processing. The present study suggests that ownership and localization of body parts may also be represented with distinct neural populations. From these findings, I propose that the processes underlying ownership and localization might be dissociated from body parts up to a whole body, thereby implying a functional distinction between “who” and “where” in the processing of formulating body information.

## Methods

### Participants

Seven male participants (mean age 26.6 years, range 22–40 years, all right-handed) with normal or corrected to normal vision participated in this study and gave informed consent in accordance with the Code of Ethics of the World Medical Association (Declaration of Helsinki). This study was approved by the Ethics Committee of the Graduate School of Information Sciences, Tohoku University.

### Apparatus and stimuli

Participants placed their right hand on a table and wore an HMD (nVisor SX60, NVIS, Reston, VA, USA; Dual SXGA microdisplays with 1280 × 1024 pixels per eye and 60° diagonal field-of-view) that displayed visual stimuli in stereoscopic 3D. The HMD was covered by black tissue to occlude all surrounding visual input and was equipped with a customized forehead rest. The arm of a PHANToM force-feedback device (3D Systems, Cary, NC, USA) was attached to the participant’s right index finger. The PHANToM can simulate haptic properties such as the friction of an object and can provide selected levels of force to the tip of the participant’s index finger. The apparatus was calibrated to spatially align the visual and haptic stimuli. Participants listened to white noise presented through headphones to mask surrounding auditory input that could have provided additional cues about the location of the haptic stimulus. In the visual-alone condition, participants viewed a realistic life-sized 3D CG hand through the HMD (Fig. 1). The luminance contrast of the CG hand varied randomly between 1.5%, 2.5%, 3.5%, 4.5%, and 5.5%. These contrasts were carefully chosen due to a preliminary examination that indicated that the effect of luminance contrast on the RHI is saturated when the contrast exceeds 5.5%. Background luminance was constant at 6.1 cd/m^2^. The colors of the CG hand and the CG cuboid were gray. Participants were instructed not to move their right hand during the experiment. In the proprioceptive-alone condition, the CG hand was not presented. The participant’s hidden right hand was passively moved by the force-feedback device. Participants were instructed to relax their hand and their muscles while being moved by the device arm and to avoid resisting the device arm.

In the visual-proprioceptive condition, participants viewed a visual sandpaper (2.5 cm × 6 cm × 0.5 cm) and either the CG hand or a 3D computer-graphics cuboid (11.5 cm × 30 cm × 3.5 cm) through the HMD (Fig. 1). The visual sandpaper moved back and forth on the index finger of the CG hand or on the CG cuboid. The force-feedback device produced an invisible haptic sandpaper (2.5 cm × 6 cm × 0.5 cm), and simulated the haptic sandpaper stroking the participant’s index finger either synchronously or asynchronously to the movements of the visual sandpaper. The visual and haptic sandpapers moved regularly in a sinusoidal manner. Motion amplitude was 2.0 cm and frequency was 1.0 Hz. In the synchronous condition, the visual and haptic sandpapers moved for 1 s sinusoidally in the same direction and amplitude and then stopped for 1 s. These synchronous movements were repeated for 60 s. In the asynchronous condition, while the haptic sandpaper was moving sinusoidally for 1 s, the visual sandpaper was stopped, after which the haptic sandpaper stopped for 1 s while the visual sandpaper moved sinusoidally for 1 s. These asynchronous movements were repeated for 60 s. The haptic stimulus was generated using the force-feedback device applied the participant’s right index finger, and appropriate forces were applied to the finger when the tip of the finger touched the simulated haptic sandpaper. Accordingly, participants felt the stroking on their index finger during the 60 s that they were exposed to the CG hand that was also being stroked on the index finger.

### Procedure

In the visual-alone or proprioceptive-alone condition, hand position discrimination was measured via a two-interval, forced-choice method. Each trial consisted of the sequential (visual or proprioceptive) presentation of two sets of horizontal hand movements. In the visual-alone condition, the CG hand was presented. The luminance contrast of the CG hand randomly varied between 1.5%, 2.5%, 3.5%, 4.5%, and 5.5% from trial to trial. In the proprioceptive-alone condition, participants reported the proprioceptive location of their right hand. In each trial, a hand started from a randomly selected location between 0 cm and 3 cm to the left of the median plane of the participant’s body. In the standard interval, the hand turned back at a distance of 5.0 cm to the right of the median plane of the participant’s body. In the comparison interval, the hand turned back at a randomly selected location between 2.5 cm and 7.5 cm to the right of the median plane of the participant’s body. The location of the hand’s turn in this interval was varied according to the method of constant stimuli. The standard and comparison stimuli were randomly assigned to the first or second interval. The proportion of trials in which the comparison stimulus was perceived to the right of the standard stimulus was then plotted. Using Probit analysis^20^, these data were fitted with cumulative Gaussian functions that provided psychometric functions (Fig. 2a). From this analysis, I obtained the mean and standard deviation of the fitted cumulative Gaussian function. The chi-square goodness of fit test showed that the curve was a good fit to the data for all conditions (χ^2^(1) = 356.6, *p* < 0.0001 for the proprioceptive-alone condition; χ^2^(1) = 162.6, *p* < 0.0001 for 1.5% contrast of the CG hand; χ^2^(1) = 255.6, *p* < 0.0001 for 2.5% contrast of the CG hand; χ^2^(1) = 285.2, *p* < 0.0001 for 3.5% contrast of the CG hand; χ^2^(1) = 392.9, *p* < 0.0001 for 4.5% contrast of the CG hand; χ^2^(1) = 480.6, *p* < 0.0001 for 5.5% contrast of the CG hand).

In the visual-proprioceptive condition, participants sat at a table and wore the HMD. At the beginning of each session, neither the CG hand nor the CG cuboid was presented on the display. Subsequently, participants were asked, “Where is your right index finger?”, and were asked to point at their own unseen right index finger by using their left hand to move a visual pointer presented in the HMD. This provided a pretest baseline estimate of finger position. After this, the CG hand or the CG cuboid was presented at a distance of 10 cm to the left of the participant’s unseen right hand. The participant’s own unseen right index finger was stroked by the haptic sandpaper for 60 s, either synchronously or asynchronously with the movements of the visual sandpaper. The contrast of the CG hand was varied randomly from trial to trial (0.5–5.5%) in the synchronous condition. The contrast of the CG hand was always 5.5% in the asynchronous condition. The contrast of the CG cuboid was always 5.5% in both conditions. After the stroking ended, participants were asked to point at their own unseen right index finger by moving a visual pointer with their left hand. Next, the participants were asked to report a subjective rating of ownership of the CG hand by pointing to a number (0 to 9) on a visual scale presented in the HMD with a visual pointer by using their left hand^34^. After all trials were complete, participants answered questionnaire items to rate subjective aspects of the CG hand. The questionnaire items for ownership were similar to those used in Botvinick and Cohen’s study^8^. Details of the questionnaire items are shown in Figure S1. Although the participants had already rated ownership for each trial on the 10-point scale, this rating included only one questionnaire item that was relevant to the illusion and did not include the questionnaire items that served as controls. For that reason, I also used the conventional 7-point scale questionnaire items to confirm that the RHI occurred in the present study.

Each participant performed 10 sessions of the synchronous condition and 10 trials of the asynchronous condition for the CG hand presentation; order was counterbalanced across participants. Each session consisted of 5 trials in the synchronous condition for the CG hand presentation, as each contrast of the CG hand (out of the five possible contrasts) was presented once a session in a random order. Each participant also completed 10 trials in both the synchronous and asynchronous conditions for the CG cuboid presentation. Order was again counterbalanced across participants.

The present study calculated the mean of the data for each participant. Through this procedure, seven samples were obtained for each experimental variable such as position and contrast. Then, these samples were averaged.

### Statistical tests

To determine whether ownership rating and perceived hand position differed among the five contrasts of the CG hand, we performed a repeated measured analysis of variance with the contrasts of the CG hand as factors. In this analysis, a single statement was used for the ownership rating (“Do you feel that the CG hand is your hand?”), which was rated on a 10-point scale for each trial. In addition, five questionnaire items (see Figure S1) were rated on a 7-point scale after all trials.

For the five questionnaire items, I compared the average of ratings on questions concerning the RHI with that of ratings on control questions across all conditions to check whether the RHI occurred, and performed a repeated measured analysis of variance with the contrasts of the CG hand and the other three control conditions as factors.

## Supplementary information

Supplemental Information

## Data Availability

The datasets generated during and/or analysed during the current study are available from the corresponding author on reasonable request.
