# Supplementary material for: Separate multisensory integration processes for ownership and localization of body parts
Source: Sci Rep. 2019 Jan 24;9:652. doi: 10.1038/s41598-018-37375-z (PMC6345910; doi:10.1038/s41598-018-37375-z)
Supplement: Supplementary file 1 — Supplemental Information [file 41598_2018_37375_MOESM1_ESM.pdf]

Supplemental Information:

Separate multisensory integration processes for ownership and localization of body parts

Matsumiya, K.

Supplemental Figure

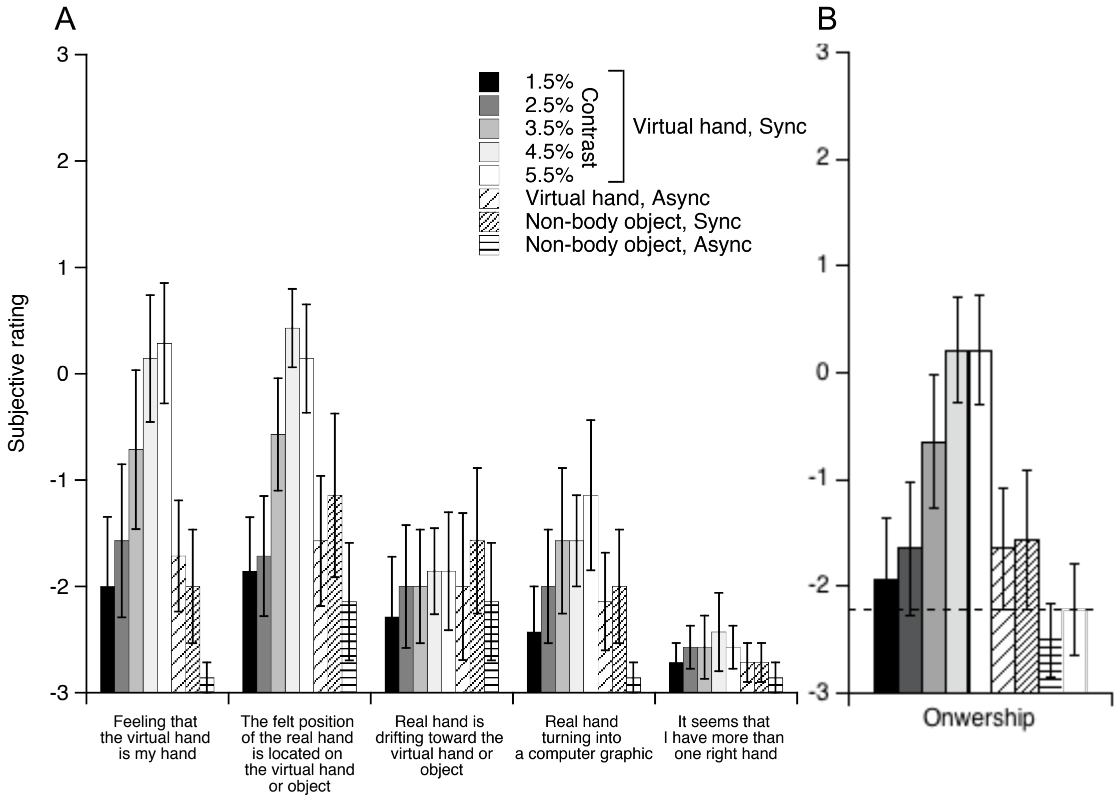

Figure S1. Subjective ratings of the rubber hand illusion. (**A**) Ownership. (**B**) Illusion questions vs control questions. Participants were asked to answer 5 questionnaire items in order to rate subjective aspects of the CG hand for the eight conditions. A positive value along the vertical axis represents agreement with the questionnaire item statements. The two questionnaire items on the left concern the illusory experience, while the others served as controls. Dotted line denotes average rating of the three control questions across all conditions. Ownership rating changed significantly depending on the conditions ($F_{8, 54}$ = 11.70, $p < 0.0001$). $n = 7$. Results are means ± standard error of the mean.

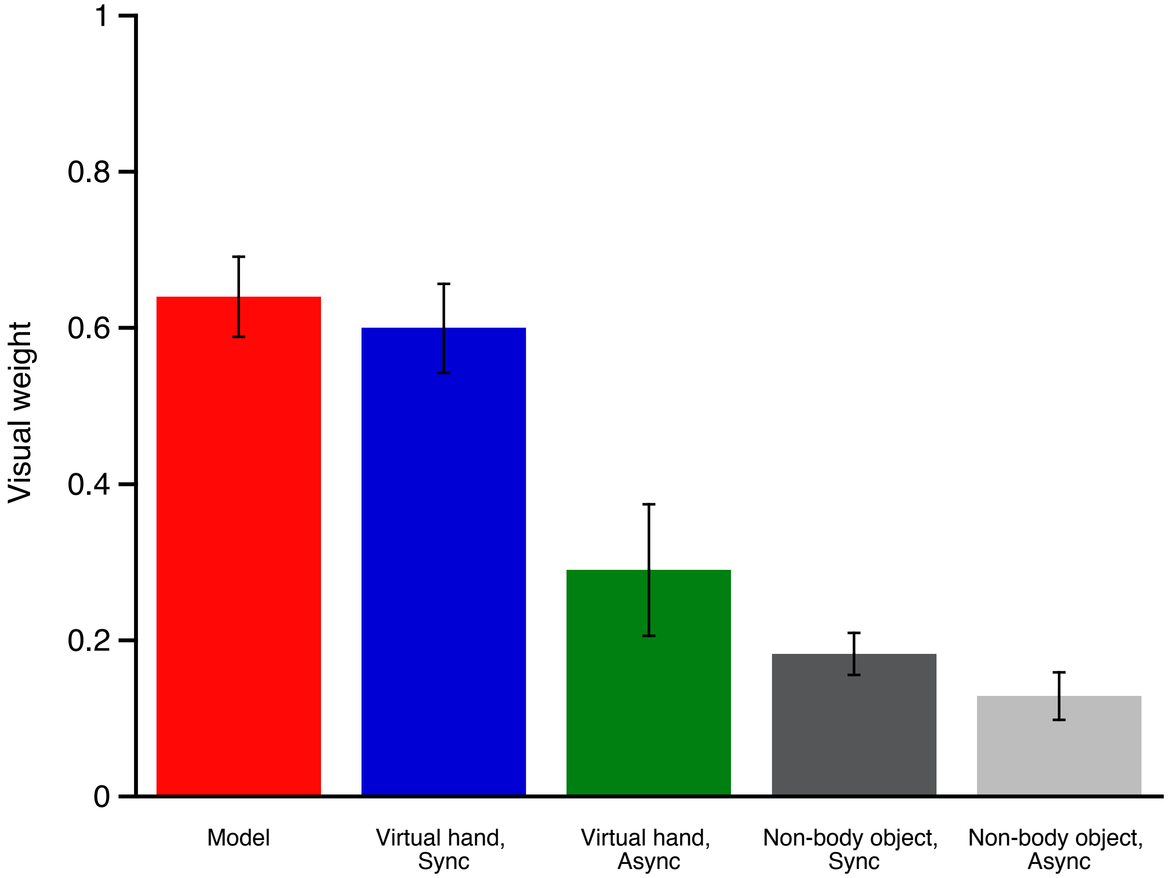

Figure S2. Visual weights. The vertical axis represents visual weight (w_v). The red bar represents the predicted weight expected from within-modality discrimination. Other bars represent observed visual weights obtained from equation 3 using the values of perceived hand positions. $n = 7$. Results are means ± standard error of the mean.
